# The Contribution of CD26-Negative Fibroblasts to Endometrial Scarring

**DOI:** 10.3390/biom15101433

**Published:** 2025-10-10

**Authors:** Muhammad Assad Riaz, Clara Marie Pecher, Franziska Louisa Kary, Jane Bosibori Maoga, Raimund Dietze, Felix Zeppernick, Ivo Meinhold-Heerlein, Lutz Konrad

**Affiliations:** 1 Center of Gynecology and Obstetrics, Faculty of Medicine, Justus Liebig University, D-35392 Giessen, Germany; muhammad.a.riaz@gyn.med.uni-giessen.de (M.A.R.); clara.pecher@outlook.de (C.M.P.); franziska.l.kary@gmail.com (F.L.K.); felix.zeppernick@gyn.med.uni-giessen.de (F.Z.); ivo.meinhold-heerlein@gyn.med.uni-giessen.de (I.M.-H.); 2 Institute of Molecular Biology and Tumor Research (IMT), Philipps University, D-35043 Marburg, Germany; raimund.dietze@web.de

**Keywords:** Menstruation, scarring, CD26, endometrium, stromal cells, fibroblasts

## Abstract

The human endometrium is unique in that it has a high potential for regeneration after menstruation without scarring. Although growth factors are thought to be responsible for scar formation, it has recently been shown for foetal skin that CD26-negative fibroblasts are essential. Thus, we investigated whether CD26 might be involved in scar formation. Primary human endometrial stromal cells (HPESCs) were stimulated with interleukin-1 alpha (IL1α) to induce CD26 protein expression, and secretion of the scar-associated proteins collagen 1 alpha 1 (COL1A1) and TGF-β3 was measured using ELISAs. The contribution of CD26 to wound closure was analysed using a wound healing assay. The CD26 inhibitor diprotin A (DPA) was used to attenuate CD26 activity. Immunohistochemistry of human uterine samples showed negligible stromal staining of CD26, but CD26 was abundant in the endometrial glands. Treatment of CD26-negative HPESCs with IL1α induced CD26 protein expression, strongly stimulated wound healing in vitro, and increased secretion of COL1A1, but decreased TGF-β3 secretion. DPA effectively attenuated all IL1α-induced effects. We suggest that the stromal non-expression of the scar-associated protein CD26 might contribute to non-scarring during endometrial wound healing.

## 1. Introduction

Menstruation is initiated by the strong decline in progesterone levels due to absence of pregnancy and only occurs in species whose endometrium spontaneously decidualize before implantation [1]. The functional layer (=functionalis) of the endometrium is broken down in a piecemeal process, shed, and subsequently restored in a non-conceptive cycle [2]. The whole process normally occurs very rapidly in the short time span of four to five days, which is only achieved by a temporary simultaneous shedding and scarless repair. During this time no cell proliferation takes place [3], but re-epithelialization and most importantly endometrial repair happen without any scarring [1]. However, the regulation and mechanisms of endometrial repair and regeneration are still poorly understood [1].

Uterine scarring occurs very rarely, mainly due to surgical interventions in the form of cesarean scar formation [4], intrauterine adhesions (IUAs) and Asherman’s syndrome [5,6] which might result in adverse pregnant consequences [7]. Cesarean scar formation is strongly and consistently associated with abnormal uterine bleeding, namely prolonged menstruation and early-cycle intermenstrual bleeding, and is three times more likely compared to healthy cases [4]. The pathological defect mainly causes postmenstrual spotting but also dysmenorrhea, dyspareunia, chronic pelvic pain, and infertility [8]. In a prospective study 90% (108/120) of patients with cesarean scar defect showed fibrosis and scar tissue [9]. In another study all cases (n = 51) demonstrated varying grades of fibrosis within the scar [10]. Histologically, cesarean scars are composed of atrophic or disorganized endometrial mucosa with regenerative epithelial atypia and fibroblastic stromal reactions but without any signs of inflammation or hemorrhage [8].

Asherman’s syndrome is characterized by endometrial fibrosis, loss of functional endometrium, obliteration of the uterine cavity by scar tissue, recurrent pregnancy loss, and infertility. Histologically, an increase in collagen deposition, increased numbers of α-smooth muscle actin-(ASMA)-positive cells, decreased numbers of glands, reduced proliferation, and reduced numbers of alternatively activated macrophages have been described [5]. Fibrotic factors, such as transforming growth factor-β1 (TGF-β1), ASMA, connective tissue growth factor (CTGF), and collagen I and III, are the key to the development of Asherman’s syndrome [6].

Investigations of endometrial scarless healing are scarce, and it was suggested that changes in stromal cell functions, mesenchymal to epithelial transition, epithelial cell proliferation and immune cells might be implicated [11]. Recently, macrophage inhibitory factor (MIF) and secretory leukocyte protease inhibitor (SLPI), two components of the menstrual fluid, inhibited collagen production, a proxy measure of scarring, in a porcine superficial wound model [12].

Mammalian tissues and organs respond to injury by scarring and fibrosis. Both are characterized by the excessive deposition of the extracellular matrix (ECM) [13], mainly collagens, resulting in scar formation during wound healing [14]. The most favorable outcome would be complete regeneration with new tissue retaining the original structural and functional attributes [13]. Fibroblasts are the crucial players that deposit ECM proteins and directly impose either scarring or scarless outcomes to injured connective tissues [13]. Scars are intrinsic cellular properties of a distinct fibroblast cell lineage or population subset, defined by CD26 expression in mouse skin fibroblasts [15]. CD26, also known as dipeptidylpeptidase 4 (DPP4), has an important role in scar formation [13,15] and fibrosis [16,17,18]. Fibrosis is found in and around the ectopic endometriotic lesions, as are myofibroblasts [19,20]. These activated fibroblasts, which result from epithelial–mesenchymal transition (EMT) or fibroblast-to-myofibroblast transition (FMT), are the most important cellular factors in fibrosis [19]. However, it is important to note that the majority of myofibroblasts (ASMA-positive fibroblasts) are localized outside the ectopic endometrial lesions [20] and partial EMT occurs more frequently after implantation than before [21].

In the human endometrium CD26 was first described to be a secretory glandular marker [22,23,24] but was later found to be expressed with medium amounts in the proliferative phase in the glands [25]. It is important to note that none of the studies [22,23,24,25] mentioned an endometrial stromal localization of CD26 or an association with scarring or fibrosis in endometriosis and adenomyosis. In the endometrium, estrogen and progesterone play important roles, and although it has been suggested that estrogen may be important in endometrial wound healing, it has been convincingly shown that estrogen is not essential for regeneration after menstruation in a mouse model [26]. Estradiol, but not progesterone, reduced the enzymatic activity of uterine CD26 in ovariectomized mice but without effects on protein concentrations [27].

Our aim was to elucidate whether CD26-negative fibroblasts could be involved in scarless endometrial wound healing. To this end, we investigated localization in the endometrium and used primary endometrial stromal cells in which we induced CD26 expression with IL1α, as published for human gingival fibroblasts [28]. Using this model, we investigated the involvement of CD26 in scar formation in vitro.

## 2. Materials and Methods

### 2.1. Human Samples

The study was approved by the Ethics Committee of the Medical Faculty of the Justus Liebig University, Giessen, Germany (registry number 95/09), and the experiments were performed in accordance with the relevant guidelines and regulations. Preoperative informed consent was obtained from all patients. All specimens were obtained by hysterectomy from patients mainly suffering from pain, leiomyomas, endometriosis or adenomyosis (Table 1) as published [29]. Dating of the endometrial tissue was based on the histological evaluation by the pathologist and the last menstrual period as reported by the patients. We used the following inclusion criteria: all women who underwent an operation of the uterus. We used the following exclusion criteria: patients with cancer.

### 2.2. Immunohistochemical Analysis and Quantification

Specimens were fixed in Bouin’s solution and embedded in paraffin. Briefly, after fixation samples were washed with 70% ethanol several times (~6 times) until the picric acid disappeared. Samples were dewaxed with Neo-Clear (Sigma Aldrich, Darmstadt, Germany) for 20 min at 40 °C and two times for 20 min at room temperature. After washing with ethanol (100%, 96%, 70%, each step two times for 5 min), the samples were washed with water and then processed further. After staining 5 µm sections with hematoxylin and eosin, the histological evaluation was performed. Serial sections of 5 µm were cut to ensure that in most cases similar areas could be examined. Immunohistochemistry was performed as published recently [30]. The EnVision Plus System (cat-no. K4002; DAKO, Hamburg, Germany) was used according to the manufacturer‘s instructions. Briefly, antigen retrieval was performed with citrate buffer (pH 6) and the jars containing the slides were put into a steamer (Multi Gourmet, Braun, Kronberg im Taunus, Germany) at 100 °C for 20 min and remained in the steamer for 20 min for cooling. Primary antibodies against CD26 (diluted 1:200, goat, cat-no. AF 1180-SP; R&D Systems, Nordenstadt, Germany), were added and incubation was done in a humidified chamber overnight at 4 °C. After washing with PBS, incubation with the appropriate secondary antibody (Anti goat labelled polymer with horseradish peroxidase, cat-no. 414161 F, Nichirei Biosciences, Tokyo, Japan) was done for 30 min at room temperature. Staining was visualized with diaminobenzidine (Liquid DAB K3467, DAKO) and counterstained with Morphisto’s hematoxylin (Morphisto, Frankfurt, Germany). After dehydration in ethanol, slides were mounted with Eukitt. Negative controls for IHC were prepared by replacement of the primary antibody by an IgG isotype (diluted 1:2000, cat-no. 02-6102, Invitrogen, Waltham, MA, USA) at the same concentration as the primary antibody. Digital images were obtained with Leica DM 2000/Leica MC170/Leica LAS 4.9.0 (Wetzlar, Germany) and processed with Adobe Photoshop CS6.

### 2.3. Isolation and Culture of Human Primary Endometrial Stromal Cells

HPESCs were isolated as described previously [31] and are CD10 positive and negative for epithelial markers such as keratin-18, keratin-19 and mucin-1. In brief, HPESCs were isolated from the eutopic endometrial tissue of a 45-year-old woman who underwent surgery by abdominal total hysterectomy due to endometriosis and uterine leiomyoma via enzymatic digestion using collagenase/dispase and maintained in complete DMEM/F12 medium (cat-no. 21041, Gibco; Darmstadt, Germany) supplemented with 10% fetal bovine serum (FCS), 1% penicillin/streptomycin (pen-strep) and 1% insulin, transferrin, and selenium (ITS) solution (complete medium) in a humidified incubator at 5% CO_2_ at 37 °C. Stromal cells at passages 4–5 were used for further analysis. All cell culture reagents were from Invitrogen/ThermoFisher Scientific (Karlsruhe, Germany).

### 2.4. Recombinant Proteins, Inhibitors, and ELISAs

The following materials were used: recombinant human IL1α (cat-no. 200-LA/CF; R&D Systems); anti-actin (cat-no. A3853, Sigma Aldrich), anti-mouse conjugated secondary antibody (cat-no. 7067) and anti-rabbit conjugated secondary antibody (cat-no. 7074) were all from Cell signaling technology (Frankfurt, Germany). 100× Halt™ Protease Inhibitor Cocktail (cat-no. P1860; ThermoFisher Scientific); Cell lysis buffer (cat-no. 9803S; Cell signaling technology); TrypLE enzyme (cat-no. 12604-021; ThermoFisher Scientific); Calcein-AM (cat-no. 425201; BioLegend; San Diego, CA, USA); Diprotin A (DPA, cat-no. ALX-260-036-M025; Enzo Life Sciences, New York, NY, USA); goat HRP-conjugated secondary antibody (cat-no. HAF109, R&D Systems), human TGF-β3 DuoSet ELISA (cat-no. DY243, range 31.2–2000 pg/mL), human COL1A1 DuoSet ELISA (cat-no. DY6220, range 31.2–2000 pg/mL) and DuoSet ELISA ancillary reagent kit 2, (cat-no. DY008B). All ELISAs are from R&D Systems.

### 2.5. Cell Culture and Experimental Protocol

All experiments were performed in complete DMEM/F12 medium containing 10% FCS, unless otherwise stated. Stromal cells were cultured at a density of 2 × 10^5^ cells per well in 6-well plates (Greiner, Kremsmünster, Austria) for 24 h. Then cells were serum-starved (1% FCS) for 24 h and treated with different concentrations of IL1α and DPA as indicated. In cases of combined treatment with IL1α and DPA, both reagents were added together. Stock solution of DPA was prepared in water and added to the respective control cells.

Treatments with hormones were conducted with charcoal-stripped FCS (cat-no. 12676-029, Gibco). HPESCs were cultured in 6-well plates and serum-starved in complete DMEM/F12 medium with 1% charcoal-stripped FCS for 24 h. Then, HPESCs were treated with 100 nM DNG (cat-no. SML1468, Sigma-Aldrich) and 10 nM E2 (cat-no. E2758, Sigma-Aldrich) alone or in combination for 48 h. A control with the diluent DMSO was used.

### 2.6. Collection of Supernatants of Cultured Cells

Cell supernatants were collected from HPESCs and centrifuged at 13,000× *g* for 15 min at 4 °C to remove detached cells. Then supernatants were transferred to tubes containing 1× Halt™ protease inhibitor cocktail and stored at −20 °C until use. For normalization, cells were washed once with 1× PBS, detached with TryplE (500 µL/well) for 5 min at 37 °C and counted after trypan blue staining using the TC20 automated cell counter (Bio-Rad, Düsseldorf, Germany).

### 2.7. Preparation of Cell Lysates and Western Blot

Culture dishes were placed on ice and cells were washed with ice-cold PBS. After aspiration of PBS, ice-cold lysis buffer containing 1× Halt™ protease inhibitor cocktail was added. The adherent cells were scraped off using a plastic cell scraper, transferred into a pre-cooled microcentrifuge tube, sonicated for 15 sec (Bandelin Sonoplus, Bandelin, Berlin, Germany) and centrifuged at 13,000× *g* for 15 min at 4 °C. Protein concentrations were determined with the Precision Red Advanced protein assay reagent (cat-no. ADV02; Cytoskeleton, Denver, CO, USA) following the manufacturer’s instructions. The Western blot was performed as previously described [31]. For detection, we used the same antibody against CD26 described in 2.2. and an anti-goat HRP conjugated secondary antibody (cat-no. HAF109, R&D Systems).

### 2.8. ELISA for Pro-Collagen 1A1 and TGF-β3

Cell supernatants were collected as published recently [32], and COL1A1 and TGF-β3 levels determined by ELISAs according to the manufacturer’s protocol.

### 2.9. Wound Healing Assay

The scratch assay was performed as described previously [32]. Briefly, 2 × 10^5^ stromal cells/well were cultured in six-well plates, and the confluent monolayer was disrupted by scratching with a sterile 200 µL pipette tip. Detached cells and debris were removed by washing twice with PBS, and cells were grown in complete medium with 1% FCS treated with IL1α (20 ng/mL) with or without DPA (20 µM). Cell migration into the cell-free areas was monitored 24 h after IL1α and DPA treatments by capturing images under a Leica DM IL microscope fitted with a Canon EOS 450D camera. Four fields were analyzed per well using ImageJ software version 1.53h (http://rsbweb.nih.gov/ij/; accessed 1 March 2021).

### 2.10. Cell Viability Assay

The cell viability assay was performed with trypan blue. Stromal cells were cultured for 24 h at a density of 2 × 10^5^ cells per well in 6-well cell culture plates. Cells were serum-starved (1% FCS) for next 24 h and then treated with IL1α (20 ng/mL) with and without DPA (0–100 µM) for 48 h. Cells were detached with TrypLE enzyme (500 µL/well) for 5 min at 37 °C and cell viability was measured after trypan blue staining using the TC20 automated cell counter (Bio-Rad) to determine the live/dead cell ratio.

### 2.11. Cell Migration Assay

Inserts with a pore size of 8 µm (ThinCert, Greiner BioOne, Frickenhausen, Germany) were placed into a 24-well plate and 1 × 10^5^ cells in 300 µL complete medium with 1% FCS were seeded on top. The lower chamber was filled with 600 µL complete medium containing 10% FCS. After 24 h at 37 °C and 5% CO_2_, medium was replaced with 450 µL serum-free medium with 8 µM Calcein-AM1 and cells incubated for 45 min in an incubator at 37 °C and 5% CO_2_. Then medium was discarded from the inserts, which were transferred into a freshly prepared 24-well plate containing 500 µL pre-warmed Trypsin-EDTA per well and incubated at 37 °C for 10 min. The inserts were discarded and 200 µL of the Trypsin-EDTA solution containing the migrated cells were transferred into a black flat bottom 96 well plate (Corning, New York, NY, USA). Fluorescence intensity was determined with the TC10 fluorescence plate reader (excitation 485 nm, emission 520 nm).

### 2.12. Statistical Analyses

Each experiment except the IHC was performed at least three times in duplicates. All data are expressed as the mean ± standard error of the mean (SEM). After testing for normal distribution with the test of Kolmogorov–Smirnov statistical comparisons of the means among multiple groups were performed by one-way analysis of variance (ANOVA). Then Dunnett’s post hoc tests were used to compare treatments with a control or the test from Tukey to compare everything with everything using the GraphPadPrism 8 software (GraphPad Inc., La Jolla, CA, USA). Differences were considered statistically significant at *p* ≤ 0.05.

## 3. Results

**Figure 1:**
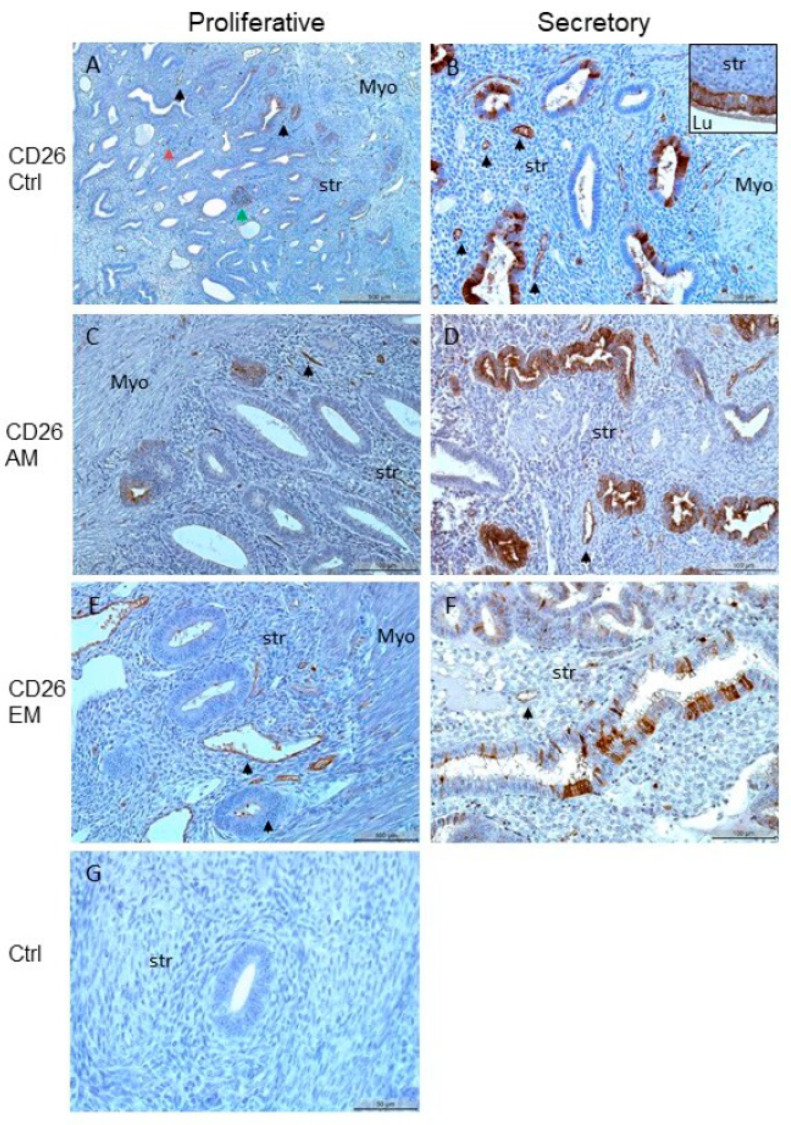
CD26 is localized in the glandular epithelial cells in proliferative (**A**) and in glandular and luminal (inset) epithelial cells in secretory (**B**) endometrium but not in the endometrial stroma. The overview shows also some plasma aggregates (green arrowhead), immune cells (red arrowhead) and blood vessels stained (**A**,**B**, arrowheads for some). CD26 is localized in the glandular epithelial cells in proliferative endometrium with adenomyosis (**C**) and with endometriosis (**E**) and in glandular epithelial cells in secretory endometrium with adenomyosis (**D**) and with endometriosis (**F**) but not in the endometrial stroma. A negative control without primary antibody is shown (**G**). Counterstaining was performed with hematoxylin. Scale bars 100 µm (**A**–**F**); scale bar 50 µm (**G**); str, stroma; lu, lumen; myo, myometrium.

Localization of CD26 could be found in the endometrial glands but not in the stroma irrespective of the cycle phase or endometrium (Figure 1A,B). The detection of the proteins in the glands was very heterogeneous, which is why we have mainly shown the stained glands in the figures in order to better contrast the unstained stroma; the blood vessels were often also CD26 positive (Figure 1). CD26 was also localized in the endometrial glands in the endometrium of women with or without adenomyosis, but not in the stromal cells (Figure 1C,D). Furthermore, CD26 was found in the endometrial glands in the endometrium of women with or without endometriosis but not in the stromal cells (Figure 1E,F). A control slide showed no CD26 staining (Figure 1G). A statistical comparison of the stromal staining was not possible because we could not identify any staining. This observation suggested that the non-expression of the scar-associated protein CD26 in the human endometrial stroma might be responsible for endometrial scarless healing.

**Figure 2:**
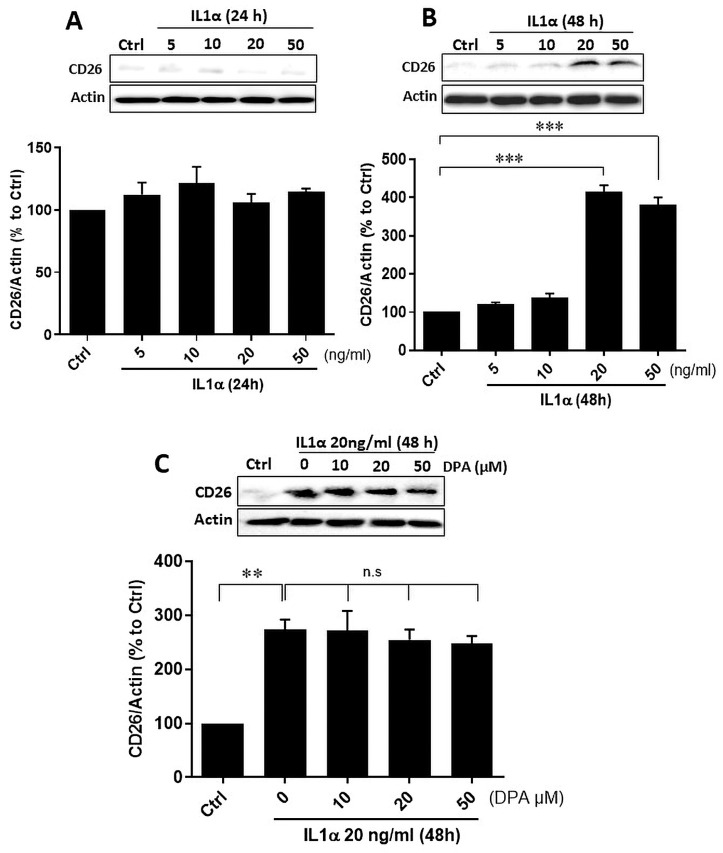
Effect of IL1α on CD26 expression in primary endometrial stromal cells. Cells were stimulated with increasing concentrations of IL1α (5–50 ng/mL) for 24 h (**A**) and 48 h (**B**) and CD26 protein levels analyzed by Western blot. Actin was used as loading control. IL1α significantly increased CD26 expression after 48 h at 20 ng/mL and 50 ng/mL. Representative immunoblots and the corresponding densitometric quantification from 3 independent experiments are shown. (**C**) Effect of IL1α and the CD26 inhibitor DPA on CD26 protein levels in stromal cells. Cells were treated with increasing concentrations of DPA and IL1α for 48 h. CD26 protein levels were analyzed by Western blot. IL1α significantly induced CD26, whereas DPA could not abrogate its effects. Unstimulated cells were set to 100% and used as a control. Each bar represents the mean ± SEM of three independent experiments performed in duplicates. ** *p* < 0.01; *** *p* < 0.001; Ctrl, control; DPA, diprotin A; n.s, not significant. Original images can be found in Supplementary Materials.

To further elucidate the importance of CD26 for wound healing, we analyzed the consequences of CD26 expression in endometrial stromal cells. Based on the observation that IL1α induced CD26 expression in human gingival fibroblasts [28], we stimulated human primary endometrial CD26-negative stromal cells with different concentrations of IL1α (5–50 ng/mL) for 24 h (Figure 2A) and 48 h (Figure 2B). IL1α treatment resulted in a concentration- and time-dependent increase in CD26 protein expression (Figure 2A,B), which was not inhibited by the CD26-specific inhibitor DPA at any concentration (Figure 2C).

**Figure 3:**
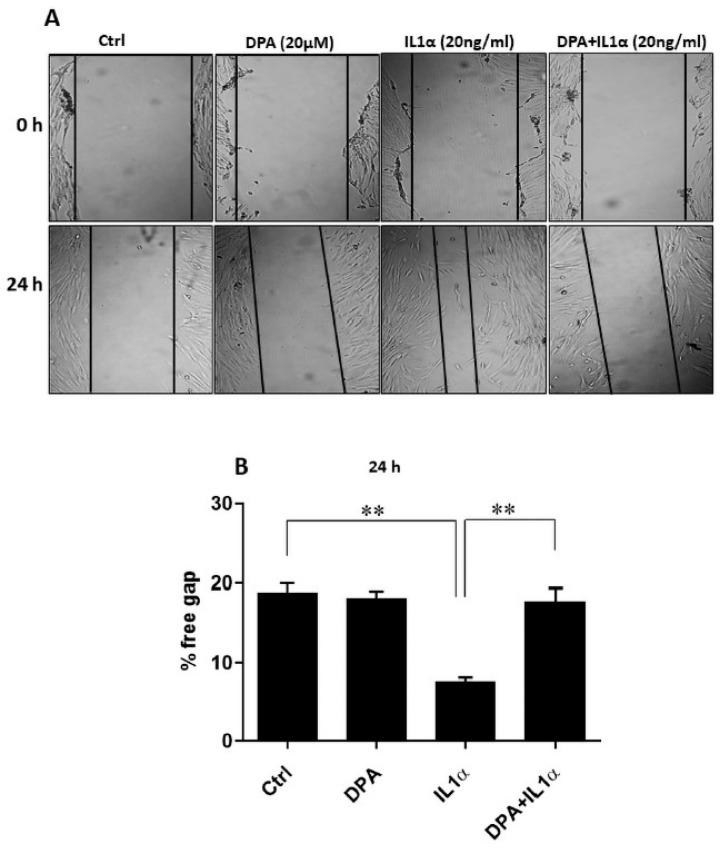
Effects of IL1α and DPA on wound healing in vitro of primary endometrial stromal cells. (**A**) The confluent monolayer of stromal cells was disrupted by scratching with a pipette tip (**upper panel**, 0 h) and incubated with DPA and IL1α. Cell-free areas were monitored 24 h after DPA and IL1α treatments by capturing images (**lower panel**, 24 h). (**B**) The results are presented as percentages of the free gaps quantified with ImageJ. IL1α promoted wound healing of the stromal cells in vitro, which was inhibited by DPA treatment. Values are presented as means ± SEM of three independent experiments performed in duplicates. ** *p* < 0.01; Ctrl, control: DPA, diprotin A.

Since estrogen and progesterone are the most important hormones in women, we also tested the effects on CD26. However, no effects of 17β-estradiol (E2) and dienogest (DNG) (alone or in combination) on CD26 protein levels were detectable in contrast to IL1α (Figure S1). Next, we investigated IL1α-stimulated wound closure of CD26-positive HPESCs in an in vitro wound healing assay and found that the IL1α-induced wound healing was clearly abrogated by DPA stimulation (Figure 3).

**Figure 4:**
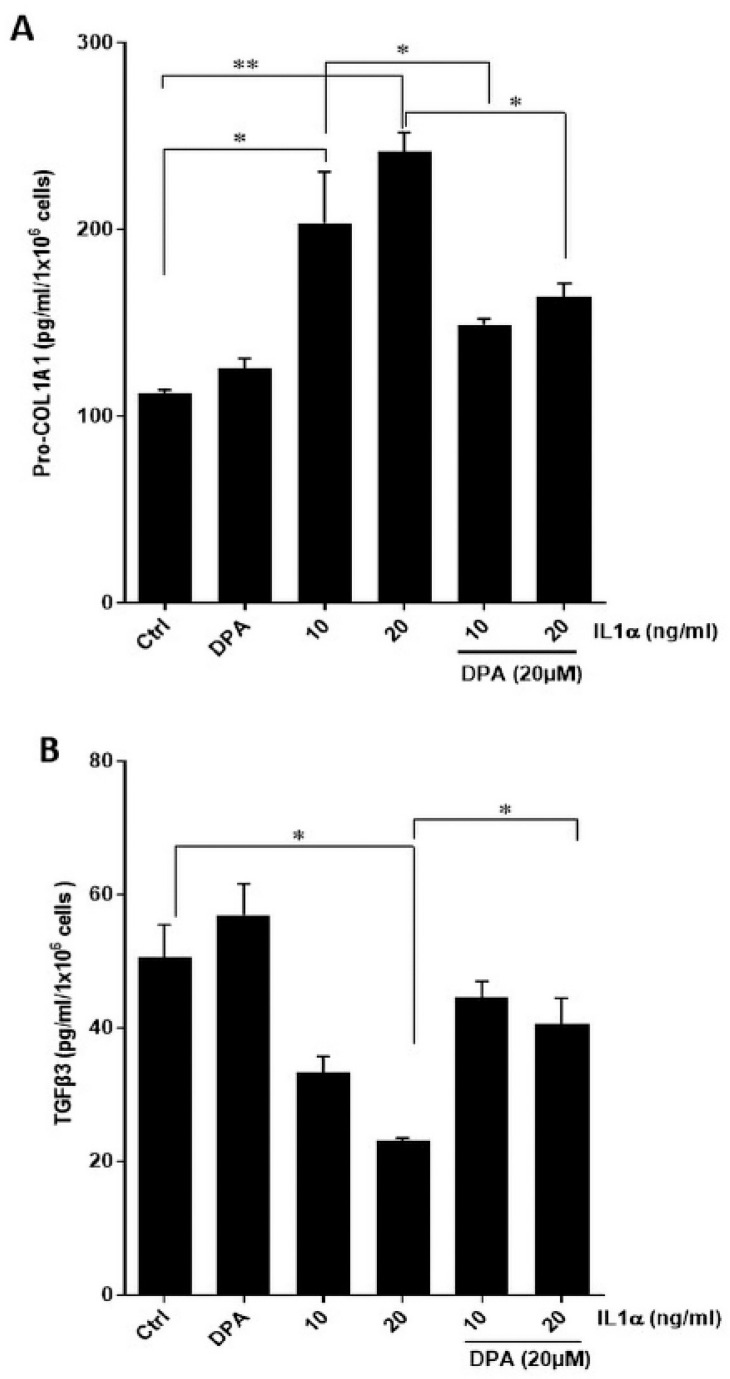
Effects of IL1α and DPA on secretion of COL1A1 and TGF-β3 of primary endometrial stromal cells. Cells were incubated with DPA and IL1α for 48 h. Secretion of COL1A1 and TGF-β3 was quantified by ELISAs. (**A**) IL1α dose-dependently increased COL1A1 secretion, which was significantly abrogated by DPA. (**B**) IL1α decreased secretion of TGF-β3, which was significantly attenuated by DPA. Untreated cells were set to 100% and used as controls. Each bar represents the means ± SEM of three independent experiments performed in duplicates. * *p* ≤ 0.05; ** *p* < 0.01; Ctrl, control; DPA, diprotin A; COL1A1, pro-collagen 1A1.

**Figure 5:**
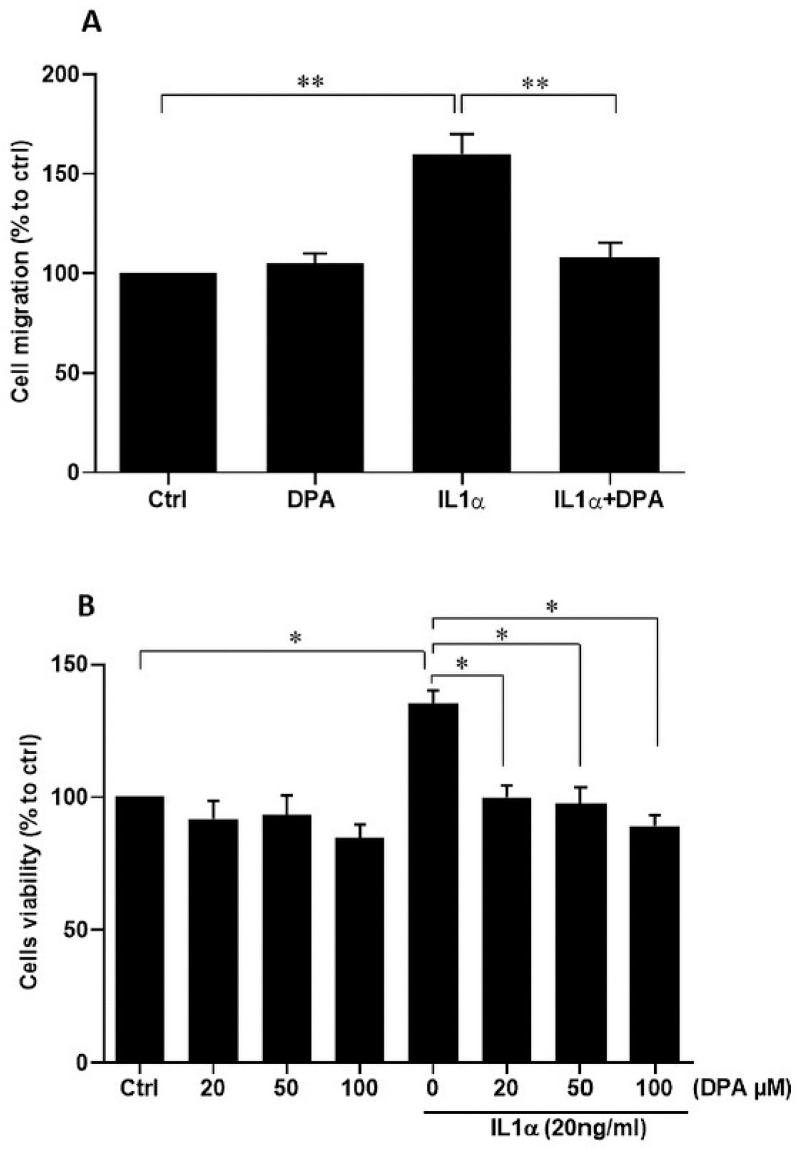
Effects of IL1α and DPA on migration and viability of primary endometrial stromal cells. (**A**) Stromal cells were incubated with DPA and IL1α for 24 h. IL1α enhanced endometrial stromal cell migration, which was inhibited by DPA. Untreated cells were set to 100% and used as a control (Ctrl). (**B**) Stromal cells were incubated with DPA and IL1α for 48 h. Cell viability was assessed by the trypan blue exclusion assay. DPA suppressed IL1α-induced cell viability. Untreated cells were set to 100% and used as a control. The values are presented as means ± SEM of three independent experiments performed in duplicates. Ctrl, control; DPA, diprotin A. * *p* ≤ 0.05; ** *p* < 0.01.

Next, we analyzed secretion of the scar-associated protein COL1A1 and found that IL1α increased COL1A1 secretion by HPESCs significantly, which was inhibited by the CD26-specific inhibitor DPA (Figure 4A). In contrast, IL1α strongly decreased secretion of TGF-β3, which was blocked by DPA treatment in HPESCs (Figure 4B). We hypothesized that IL1α-stimulated wound healing might be caused by increased cell migration, which was indeed the case (Figure 5A). The IL1α-increased cell migration was strongly reduced by DPA (Figure 5A). Furthermore, IL1α also increased stromal cell viability that was also efficiently suppressed by DPA (Figure 5B).

## 4. Discussion

Our experiments suggest that the absence of CD26 expression in the endometrial stroma may be at least partially associated with scar-free healing during menstruation. To further support this hypothesis, we induced CD26 expression in primary endometrial stromal cells and observed a significant effect on the scar-associated proteins COL1A1 and TGF-β3. Both effects could be inhibited by the CD26-specific inhibitor DPA. Finally, CD26 accelerated wound healing in an in vitro wound healing assay, which was attenuated by DPA.

Fibroblasts are the crucial players that deposit ECM proteins and thus are responsible either for scarring or scarless outcomes of injured tissues [13]. Thus, fibroblasts are one of the most important targets for scarless wound healing [33]. Scars are intrinsic cellular properties of a distinct fibroblast cell lineage or population subset, defined by CD26 expression in mouse skin fibroblasts [15]. Most often CD26 is a marker of activated fibroblasts which deposit excessive ECM, mainly collagens, during wound healing leading to scar formation [13]. In the human endometrium, CD26 is localized in the glandular epithelium but not in the stromal cells [22,23,24,25], but none of the authors provided information on whether the patients had endometriosis and/or adenomyosis. We were able to confirm the endometrial epithelial localization and the lack of localization in endometrial stromal cells in our study and additionally showed that neither endometriosis nor adenomyosis had any influence on the lack of CD26 expression in the endometrial stromal cells.

Although the missing endometrial stromal expression of the scar-associated protein CD26 was a strong indication for the involvement in scarless endometrial healing during menstruation, conclusive evidence was lacking. Thus, we decided to induce expression of CD26 in isolated HPESCs. Treatment of CD26-negative endometrial stromal cells with IL1α considerably increased CD26 expression resulting in increased scar-associated COL1A1 secretion but decreased TGF-β3 secretion, which is well known for its involvement in fetal scarless healing (reviewed in [13]). Furthermore, CD26-positive HPESCs showed an increased wound closure in an in vitro wound healing assay. The specific CD26 inhibitor DPA could attenuate all these effects of CD26-positive endometrial cells. The increased wound closure is possibly due to increased CD26-dependent cell migration as shown in endometrial cancer [34]. In human skin CD26 is expressed in a subpopulation of fibroblasts that synthesize the majority of COL1A1 during wound healing [35]. Similarly, human CD26+ fibroblasts produced more ECM proteins, fibronectin and collagen I, both in vitro and in vivo during regeneration of transplanted skin compared to CD26+ fibroblasts [36]. In this context, it is also interesting to note that CD26 inhibitors attenuated both migration and fibrosis of CD26-positive fibroblasts [37,38].

It is now assumed that CD26 attenuates fibrotic markers such as TGF-β, pSmad2/3, ASMA, COL1, COL3, and downstream effectors of the MAPK-NF-κB pathway in many wound healing models [39]. In this respect, initial applications of zinc sulfide cellulose nanofiber scaffolds with the CD26 inhibitor sitagliptin showed a significant reduction in scarring during wound healing and reduced fibrosis of the skin in rats [40]. Similar approaches could also be used for fibrosis in endometriosis lesions or scarring after cesarean sections, intrauterine adhesions, or Asherman’s syndrome.

In another approach, menstrual fluid (MF), which promotes the healing of endometrial and keratinocyte “wounds” in vitro, was investigated for its re-epithelialization properties in an in vivo model for pig wounds. Proteome analysis of MF identified a large number of proteins such as migration inhibitory factor, neutrophil gelatinase-associated lipocalin, follistatin-like protein-1, chemokine ligand-20, and secretory leukocyte protease inhibitor. All of these proteins were selected for further investigation and were shown to repair endometrial and keratinocyte wounds by promoting migration [12]. These approaches show that wound healing is a complex process and depends on several factors. There are many studies on IL1 in endometriosis, especially on IL1β, but very few on IL1α. Even though it is assumed that pro-inflammatory cytokines are also expressed at elevated levels in the eutopic endometrium of women with endometriosis, no difference was found for IL1α compared to healthy women [41]. Furthermore, IL1α was nearly exclusively found in the glands and mostly with a weak to absent expression [41]. In contrast, IL1α but also its antagonist interleukin-1 receptor antagonist (IL1RA), are present in increased amounts in peritoneal fluid of women with endometriosis vs. without endometriosis [42], but IL1RA is not found in endometriotic lesions [43]. This could be a possible explanation for the absence of IL1-dependent (among other factors) fibrosis in the endometrium, but its occurrence in endometriotic lesions. It would also be a plausible hypothesis as to why IL1α does not induce CD26 in the endometrium in vivo but does so in isolated stromal cells. Another possibility would be the blockade of CD26 gene transcription through methylation [44]. Future experiments will have to show which of these hypotheses are correct.

### Strengths and Limitations

The strength of this study lies in the fact that, for the first time, it has been possible to demonstrate with a high degree of probability that the absence of scar-associated fibroblasts could be connected to scar-free wound healing in the endometrium. Although we used fibroblasts from a woman with endometriosis, this does not invalidate the study, as we found no evidence in immunohistochemistry that endometrial fibroblasts from women with/without endometriosis and with/without adenomyosis differ in terms of CD26 protein expression. Furthermore, we recently found that endometrial RNA expression without/with endometriosis differs by only 0.92% [45].

It is conceivable that other factors such as for example Yes1 associated protein1 (YAP1) could also play a role in scar-free wound healing in the endometrium, as the transcription factor YAP1 is a key signal in skin fibroblasts in the fibrotic response and thus in scar formation [46]. Mechanically induced scars showed the highest YAP1 expression, which upregulates CD26, engrailed-1 (En-1) and collagen deposition. Blocking YAP1 prevented En-1 activation in skin fibroblasts, resulting in wound healing without scar formation [46]. In the human endometrium, one study found expression in the stroma and glands [47], while another study found localization only in the glands [48]. In initial experiments, we also found localization of YAP1 primarily in the endometrial glands. Similar experiments to those conducted in this study could shed light on the role of YAP1 in scar-free endometrial wound healing.

## 5. Conclusions

We have clarified the strongly restricted endometrial epithelial expression of the scar-associated protein CD26 in the human endometrium irrespective of endometriosis or adenomyosis. We have clearly shown that the missing endometrial stromal expression of CD26 is likely and with a high probability associated with endometrial scarless healing during menstruation. Further experiments could reveal whether induced CD26 expression in endometrial stromal cells contributes to scarring in cesarean scar formation, intrauterine adhesions or Asherman’s syndrome. We anticipate that our findings will stimulate further research into scarless endometrial healing and fibrosis.

## Figures and Tables

**Table 1:** Overview of the endometrial tissue samples.

Tissues	EM-P	EM-S	*p* Value
All samples (n)	25	30	
Age ± SD	41.1 ± 8.0	40.3 ± 5.5	n.s.
Adenomyosis	13	12	
Endometriosis	14	16	
Leiomyoma	10	13	

EM-P, proliferative endometrium; EM-S, secretory endometrium; SD, standard deviation; n.s., not significant.

## Data Availability

All data are available from the corresponding author upon request.

## Supplementary Materials

The following supporting information can be downloaded at: https://www.mdpi.com/article/10.3390/biom15101433/s1, Figure S1 and original images of western blot.
