# Supplementary material for: The Contribution of CD26-Negative Fibroblasts to Endometrial Scarring
Source: Biomolecules. 2025 Oct 10;15(10):1433. doi: 10.3390/biom15101433 (PMC12563657; doi:10.3390/biom15101433)
Supplement: Supplementary file 1 [file biomolecules-15-01433-s001.zip › biomolecules-3867708-supplementary.pdf]

Figure S1

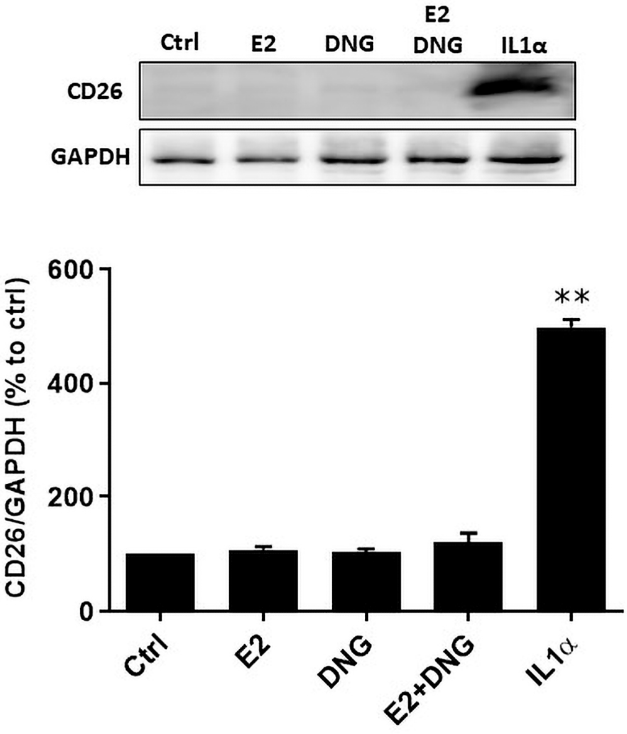

Figure S1. Effects of 17β-estradiol (E2, 10 nM), dienogest (DNG, 100 nM), E2/DNG combined and IL1α (20 ng/ml) on CD26 in primary endometrial stromal cells HPESCs. Cells were stimulated for 48 h and CD26 protein levels analyzed by Western blot. GAPDH was used as loading control. Only IL1α significantly increased CD26 expression. Unstimulated cells were set to 100% and used as a control. Representative immunoblots and the corresponding densitometric quantification from 3 independent experiments are shown. Each bar represents the mean ± SEM of three independent experiments performed in duplicates. ** $P < 0.01$; Ctrl, control; E2, 17β-estradiol; DNG, dienogest.
